# Effects of Intake of Maternal Dietary Elaidic Acids during Pregnancy and Lactation on the Fatty Acid Composition of Plasma, Erythrocyte Membrane, and Brain in Rat Pups

**DOI:** 10.1155/2013/701818

**Published:** 2013-09-30

**Authors:** Noriko Komatsuzaki, Ayumi Eda, Rie Kameoka, Yoko Nakashima

**Affiliations:** Department of Human Nutrition, Seitoku University, 550 Iwase, Matsudo, Chiba 271-8555, Japan

## Abstract

To investigate the effects of a dam's dietary elaidic acid (EA) intake during pregnancy and lactation on the fatty acid composition of plasma, erythrocyte membrane, and brain in rat pups, we fed two groups of dams either a soybean oil diet (SOD) or a shortening diet (SHD) containing soybean oil (10%) or shortening (10%), respectively. Although EA was not detected in the SOD, EA accounted for 25.3% of all fatty acid content in the SHD. On day 8 after birth, the EA levels in the stomach, plasma, and erythrocyte membrane of pups nursed by the dams fed the SHD were 11.6 ± 1.03%, 7.18 ± 1.20%, and 5.82 ± 1.00%, respectively. Although on day 8 after birth the EA level of the brains of pups nursed by SHD-fed dams was 0.56 ± 0.24%, EA was not detected on day 21 or day 82 after birth. These results suggest that EA intake during pregnancy and lactation supplies EA to plasma, remains in the erythrocyte membrane of pups, and moves into the brain in early infancy.

## 1. Introduction

Trans fatty acids (TFAs) are created from vegetable oils through hydrogenation. In the trans configuration, the carbon chain extends from opposite sides of the double bond, rendering a straight molecule. Elaidic acid (EA; C_18:1_) is the principal TFA often found in partially hydrogenated vegetable oils, margarine, and shortening [1, 2]. Young people in Japan have come to prefer a Western-style diet, and now more than 29% of the calories consumed by Japanese people are provided by fat [3]. The consumption of trans fats increases the risk of coronary heart disease (CHD) by raising the levels of LDL cholesterol and lowering the levels of HDL cholesterol [2, 4, 5]. It has been reported that an elevated TFA blood cell content increased the risk of acute coronary syndromes (ACS) [6, 7].

Infants are exposed to TFA before and after birth by the transfer of fatty acids originating from the maternal diet [9]. On average, mature human milk provides 3.7 g fat/100 mL, representing about 50% of the dietary energy intake of the young infant [10, 11]. Infants receiving human milk ingest levels of TFAs and essential fatty acids that reflect short-and long-term maternal diets [12–14]. Recent studies have indicated that the EA content of the maternal diet may be associated with both maternal and infant body composition in the early postpartum period [15]. 

We previously reported that dietary fish oil intake during pregnancy and lactation provides more n-3 polyunsaturated fatty acids (PUFA) to pups and alters their brain fatty acid composition [16]. However, it has been reported that prenatal essential fatty acid deficiency may result in myelin with an abnormal composition and structure, at least during the critical period of brain development [17]. Trans-enriched isomers of *α*-linolenic acid (ALA) were fed to female adult rats during gestation and lactation. The *trans* ALA can be desaturated and elongated *in vivo* and thus provides *trans* isomers of the n-3 fatty acids eicosapentaenoic acid (EPA) and docosahexaenoic acid (DHA), even in pup brain structures [18]. 

Animal studies have demonstrated that brain DHA concentrations reduced after relatively high intakes of EA [19]. Thus, a dam's dietary EA intake during pregnancy and lactation may affect the development of the brain of her pups. Although *trans* ALA was reportedly absorbed in the rat brain, there are no reports on the effects of dietary EA on the brain fatty acid composition of pups, to the best of our knowledge. The aim of the present study was to determine whether a dam's dietary EA intake during pregnancy and lactation would affect the plasma, erythrocyte, and brain fatty acid composition in pups. We investigated the effects of the maternal ingestion of a shortening diet (SHD) and a soybean oil diet (SOD) on 82-day-old offspring. Body and fat tissue weights as well as plasma lipid concentrations were alsomeasured, as was the growth of the pups.

## 2. Materials and Methods

### 2.1. Animals and Diets

Ten sperm-positive pregnant rats of the Sprague-Dawley strain on day 3 of pregnancy (10 weeks old) were commercially obtained from Clea Japan (Tokyo). They were housed individually in plastic cages with paper chip (ALPHA-DriTM, Shepherd Specialty Papers, Chicago, IL) bedding. They were maintained in a room kept at a constant temperature (22 ± 1°C) and 50% relative humidity with a 12 h dark/light cycle (19:00–7:00). They had free access to food and water. They were weighed, and their food intake was measured every day between 16:00 and 18:00 during the experimental period.

The composition of each diet is shown in Tables 1 and 2. The SOD and SHD were based on the AIN-93G diet [8]. The diets were prepared by adding soybean oil (10%) and shortening (10%), respectively, to the AIN-93G, substituting for cornstarch. Soybean oil and dietary components were obtained from Oriental Yeast Co. (Tokyo). Shortening was purchased from Kaneka Co. (Osaka, Japan). Soybean oil, the Triglyceride *E*-test Wako, the Cholesterol *E*-test Wako, and other chemicals were purchased from Wako Pure Chemical Industries (Osaka).

### 2.2. Experimental Design

Ten pregnant rats (on day 3 of pregnancy) were divided into two equal groups. One group was fed the SOD and the other group the SHD during the experimental period (pregnancy and lactation). Within 24 h of birth, each litter was culled to 10 pups (5 males and 5 females) and nursed by their dams for 21 d. On days 8 and 21 after birth, two pups (a male and a female) from each litter were killed by decapitation and their blood was collected. The blood was collected in standard evacuated tubes using EDTA as an anticoagulant. Whole blood was centrifuged for 15 min at 1400 ×g, and plasma was aliquoted into microcentrifuge tubes. The buffer layer of white blood cells was removed using a pasture pipette, and then the erythrocytemembrane was aliquoted into separate microcentrifuge tubes. Blood was also collected into one tube without EDTA to provide serum. Perirenal fat tissue and liver were removed and weighed.

After weaning (at 21 d of age), the remaining pups were housed individually. The pups were fed the same diet regardless of group. On days 47, 68, and 82 after birth, the pups were fasted for 16 h and sacrificed without affliction under ether anesthesia for collection of the liver, perirenal fat tissue, and brain. The plasma, liver, and brain were stored at −80°C until they were analyzed.

All procedures were performed in accordance with the Animal Experimentation Guidelines of the Laboratory Animal Care Committee of Seitoku University.

### 2.3. Biochemical Assays of Plasma, Liver, and Brain

Liver and brain lipids were extracted by the method of Folch et al. [20]. Triacylglycerol (TG) and total cholesterol (T-cho) concentrations in plasma as well as liver extracts were measured using test kits (Triglyceride *E*-test Wako, Cholesterol *E*-test Wako).

### 2.4. Fatty Acid Compositions of Plasma, ErythrocyteMembrane, and Brain

Packed erythrocytemembranes were diluted in 500 *μ*L of water and thoroughly mixed by vortex, and the mixture was kept on ice for 15 min. To initiate the lipid extraction, isopropanol (4 mL) was added, and the samples were vigorously mixed by vortex and kept for 1 h with frequent mixing. Then hexane (6 mL) was added, followed by frequent mixing for another hour [21]. The samples were subjected to centrifugation at 1200 ×g for 10 min, and the lipid-containing organic fraction was removed. 

Fatty acid in the plasma, erythrocytemembrane, and brain extract was methylated according to the procedure of Morrison and Smith [22]. First, 1.5 mL of 0.5 mol NaOH dissolved in methanol was added to 0.2 mL of plasma, and the solution was subjected to vortex mixing and then boiled in a water bath at 100°C for 9 min. Next, 2 mL of boron trifluoride methanol complex methanol solution was added to the mixture, followed by vortex mixing and incubation in a water bath at 100°C for 7 min. After cooling, *n*-hexane (3 mL) was added to the mixture, which was shaken well. The saturated NaCl solution (5 mL) was added to the mixture during vortex mixing, followed by centrifugation at 1500 ×g for 10 min. The *n*-hexane phase containing the fatty acids methyl esters (FAMEs) was extracted. The FAMEs were analyzed by a Hitachi G-3500 gas chromatograph (GC) fitted with a split injector (250°C) and a flame ionization detector (260°C) coupled with a chromatointegrator (Hitachi D-2500). We used a TC-70 column (0.25 mm ID × 60 m length, 0.2 *μ*m film thickness; GL Sciences, Tokyo), with a helium (He) carrier gas flow rate of 1 mL/min. The column was isothermally kept at 190°C for 15 min, heated from 190°C to 200°C at a heating rate of 5°C/min, kept at 200°C for 10 min, heated from 200°C to 210°C at a heating rate of 5°C/min, and kept at 210°C for 4 min. Each FAME peak was identified with a standard FAME (Funakoshi Co., Tokyo).

### 2.5. Statistical Analysis

Values are expressed as the mean ± SD. Student's *t*-test was used for all pairwise comparisons. Differences in values between groups were tested by Scheffe's multiple-range test. A *P* value of less than 0.05 was considered significant. 

## 3. Results 

### 3.1. Food Intake, Body, Liver, and Perirenal Fat Tissue Weights, and Plasma Lipid Concentrations in Dams

During pregnancy and lactation, no significant difference in the total food intake of the dams was observed between the SOD and SHD groups. No significant difference in body, liver, or perirenal fat tissue weights was observed between the two groups (Table 3). No significant difference was found in total litter size between the groups (13.2 ± 2.49 and 11.0 ± 4.64). The male and female litter sizes in the SOD group were 7.00 ± 2.24 and 6.20 ± 1.10, respectively, whereas those in the SHD group were 6.20 ± 1.10 and 4.60 ± 2.19, respectively. Therefore, the two maternal diets did not affect the litter size or the ratio of female versus male pups. Within 24 h after birth, the litters were weighed. The pups nursed by dams fed the SOD and SHD weighed 7.40 ± 0.18 g and 7.27 ± 0.34 g, respectively, indicating no significant difference between the groups.

No significant differences in plasma TG or T-cho concentrations in the dams were observed between the two groups (Table 3).

### 3.2. The Fatty Acid Composition of Stomach Content in 8-Day-Old Pups Nursed by Dams Fed SOD or SHD

Table 4 shows the fatty acid composition of stomach content in 8-day-old pups nursed by dams fed SOD and SHD. The level of saturated fatty acids in the stomach content was the highest in both groups, and no significant difference was observed between the groups. The monounsaturated fatty acid in the stomach content of the SHD group was significantly higher than that of the SOD group (*P* < 0.05). The n-6 and n-3 fatty acids in the stomach content of the SOD group were significantly higher than those of the SHD group (*P* < 0.01). The EA in the stomach contents of the SHD group was 11.6 ± 1.03, but EA was not detected in the stomach contents of the SOD group. The ratios of n-6/n-3 of the SOD and SHD groups were 15.1 and 54.7, respectively.

### 3.3. Body, Liver, and Perirenal Fat Tissue Weights and Plasma Lipid Concentrations of Pups Nursed by Dams Fed the SOD or SHD 

On days 8, 21, 47, 68, and 82 after birth, no significant differences in body, liver, or perirenal fat tissue weights were observed between the groups (Table 5). The liver and perirenal fat tissue weights of the pups of both groups increased with growth. 

On days 8 and 21 after birth, the plasma TG and T-cho concentrations in the pups of both groups were high but decreased after weaning (Table 6). No significant difference in plasma TG concentration was observed between the groups. On day 21 after birth, the plasma T-cho concentration of the SHD group was significantly lower than that of the SOD group (*P* < 0.05). However, on day 47 after birth, no significant difference in the plasma T-cho concentration was observed between the groups.

### 3.4. Fatty Acid Compositions of the Plasma and Blood Cell Membrane of Pups

On days 8 and 21 after birth, the saturated, n-6, and n-3 fatty acids were significantly higher in the plasma of the SOD group than in that of the SHD group (*P* < 0.05) (Table 7). On the other hand, the monounsaturated fatty acid in plasma was significantly higher in the SHD group than in the SOD group (*P* < 0.05). EA was detected in the plasma of the SHD group but not in that of the SOD group. The EA in the plasma of the SHD group on day 21 after birth was significantly higher than that on day 8 after birth (*P* < 0.05). On day 8 after birth, compared with the fatty acid composition of the stomach contents and the plasma, the saturated fatty acid in plasma was decreased in both groups, whereas the n-6 and n-3 fatty acids were significantly increased in the plasma of both groups (*P* < 0.05).

Although no significant difference in the saturated fatty acid in the erythrocyte membrane of the pups on days 8 and 21 after birth was observed between the groups, the n-6 and n-3 fatty acids in the erythrocyte membrane were significantly higher in pups of the SOD group than in those of the SHD group (*P* < 0.05) (Table 7). On days 8 and 21 after birth, the monounsaturated fatty acid in plasma and blood cell membrane was higher in the SHD group than in the SOD group (*P* < 0.05) (Table 7). The EA in the erythrocyte membrane of the SHD group was significantly higher on day 21 after birth than on day 8 after birth (*P* < 0.05). In the SHD group on days 8 and 21 after birth, the EA was significantly higher in plasma than in erythrocyte membrane, and n-3 fatty acid levels were significantly higher in erythrocyte membrane than in plasma (*P* < 0.05).

### 3.5. Fatty Acid Composition in Pup Brains

The brain weight (g/100 g BW) of pups of both groups decreased significantly with growth (*P* < 0.05) (Table 8). The total fat (mg/g brain) in the pup brains of both groups increased significantly with age (*P* < 0.05). However, on days 8, 21, and 82 after birth, no significant differences in the brain weight and total fat were observed between the groups. The saturated fatty acid in the brains of pups was 50%. Although no significant difference was found between the groups in the level of saturated fatty acid in the brains of pups on day 8 after birth, on day 21 the SHD group had significantly higher levels than the SOD group (*P* < 0.05). Although the level of monounsaturated fatty acid in the brains on day 8 after birth was higher in the SHD group than in the SOD group (*P* < 0.05), no significant differences were observed between the groups at 21 and 82 days after birth. The monounsaturated fatty acid levels in the brains of pups of both groups decreased with growth (*P* < 0.05) (Table 8). Although no significant difference between the groups was observed in the n-6 fatty acid in brains of pups on day 8 or day 21 after birth, on day 82 the SHD group had higher levels than the SOD group (*P* < 0.05). Although on days 8 and 21 after birth n-3 fatty acid levels were lower in the brains of the SHD pups than in those of the SOD pups (*P* < 0.05), no significant difference was observed between the groups at 82 days. EA was not detected in the brains of SOD group pups. Although the EA of the brains of pups of the SHD group on day 8 after birth was 0.56 ± 0.24%, EA was not detected on day 21 or day 82. On days 8, 21, and 82 after birth, the n-6/n-3 ratio in pup brains was higher in the SHD group.

## 4. Discussion 

On day 8 after birth, the saturated fatty acid composition of the stomach contents of pups of both groups was higher than that of the dams fed either diet. This indicated that a large amount of saturated fatty acid was synthesized in the dam body in order to produce high-fat milk. Grigor et al. reported that the specific activity of lipogenic enzyme was higher in the mammary gland and liver of lactating rats than in those of virgin animals [23]. On the other hand, level of monounsaturated fatty acid, as well as of n-6 and n-3 fatty acids, was lower in the stomachs of pups in both groups than the levels of the same fatty acid composition of either diet. We considered that this phenomenon happened because of the increased ratio of saturated fatty acid in the stomachs of the pups.

Kavanagh et al. fed female C57/BL6 mice a trans diet containing hydrogenated vegetable oil during pregnancy and lactation and investigated the fatty acid composition in their breast milk [24]. The EA (18:1 n-9) in the trans diet and breast milk was 26.9% and 11.9%, respectively. In the present study, EA in the SHD was 25.3%, and in the stomach content of the SHD group on day 8 after birth it was 11.6 ± 1.03% (Table 4); these data are mostly consistent with the results of Kavanagh et al. Moreover, those authors reported that although the n-3 fatty acid was lower in the EA diet than in the control diet, the n-3 fatty acid levels in breast milk were almost the same between the groups [24]. In our previous report, pregnant rats were fed a corn oil diet or a perilla oil diet, and their fatty acid levels in breast milk were compared [25]. We reported that n-3 fatty acid was preferentially provided to breast milk when the dams were fed the n-3 fatty acid-deficient diet; the ratio of n-6/n-3 in the breast milk in that group was lower than that in dams fed the other diet [25]. In the present study, the ratios of n-6/n-3 in SHD and in the stomach content of the SHD group were 64 and 54.7, respectively. The ratio of n-6/n-3 in stomach content of the SHD group was lower than the ratio of n-6/n-3 in the SHD group, similar to the results of the report of Kavanagh et al.

There are several reports on the effects of TFA intake during pregnancy and lactation on fetuses and infants. Innis et al. indicated that a low intake of essential fatty acids during pregnancy lowed fetal development and that it was possible that TFA accumulated in the plasma of fetuses [10, 26]. In the present study, TFA intake during pregnancy and lactation showed no effect on birth weight or growth after birth (Table 5). Although no significant differences in plasma TG and T-cho concentrations were observed between the groups, the plasma T-cho concentration in pups on day 82 after birth tended to be higher in the SHD group (*P* = 0.062) (Table 6). It was reported that TFA intake during pregnancy and lactation increased LDL-cholesterol levels in plasma and induced changes in maternal and infant body composition and the development of obesity later in childhood [5, 15]. It is possible that the plasma concentration in pups of the SHD group was changed after 82 days of age. Studies that follow rats throughout their lifetimes are needed.

Compared with the plasma fatty acid composition of pups between 8 and 21 days old, the EA in plasma of the SHD group on day 21 after birth was approximately twice that on day 8 after birth (Table 7). The pups were fed only breast milk until day 8 after birth. Although the pups were weaned on day 21 after birth, they were fed breast milk and SHD. Therefore, the pups of the SHD group on day 21 after birth were fed a large quantity of EA in the SHD, and this caused the EA to increase in the plasma of pups in that group. The EA in the erythrocyte membrane of pups of the SHD group on day 21 after birth was higher than that of pups at 8 days old, the same pattern that was found in plasma (Table 7).

Although the n-6 and n-3 fatty acids in the erythrocyte membrane were lower in the SHD group than in the SOD group, the ratio of n-6/n-3 in erythrocyte membrane of the SHD group was higher on days 8 and 21. Block et al. reported that, compared to controls, the blood cell membrane content of n-3 PUFA was lower in ACS cases; levels of TFA and arachidonic acid (AA; 20:4 n-6) were higher [6]. AA is found in cell membrane phospholipids and is important in infant growth and central nervous system development [27]. In a recent study, significant positive relationships were found between TFA level in erythrocyte membrane and C-reactive protein (CRP), suggesting that the TFA intake might be a predictor of increased risk for metabolic syndrome [28]. During pregnancy and lactation in the present study, TFA was provided in breast milk and increased the TFA levels in erythrocyte membrane of SHD group pups. These results indicated that TFA intake during pregnancy and lactation threatened the health of pups. 

From days 8 to 21 after birth, the rates of increase in the total fat in pup brain were 2.6 times and 2.3 times in the SOD and SHD groups, respectively. This indicated increased lipid and energy supplied to the brain for its rapid development. Previously, we reported on the effects of a deficiency of n-3 fatty acid during pregnancy and lactation on the composition of fatty acid in the brains of rat pups [16]. Whereas fish oil contains high levels of n-3 fatty acids, such as EPA and DHA, there are no fatty acids in lard. Although levels of n-3 fatty acids in the brains of pups nursed by dams fed fish oil during pregnancy and lactation were higher than the levels in a group whose dams were fed lard, there was no significant difference in the ratio of n-3/n-6 in the brains of pups between the groups. In this study, the ratios of n-6/n-3 in the brains of pups of the SHD group on days 8, 21, and 82 were 1.6, 1.3, and 1.1 times that of the SOD group, respectively, and the fatty acid composition of the brains of pups remained constant (Table 7). Although the EA in SHD dams taken during pregnancy and lactation was 25.3%, the ratio of EA decreased as the EA moved into breast milk, plasma, and blood cells and brains of pups on day 8 after birth. n-3 fatty acids are not consumed as a source of energy because of their poor intake; these acids are used in the composition of the body. On the other hand, it is supposed that the EA is used as a source of energy with oleic acid (C_18-1_), which is contained abundantly in SHD.

EA was not detected in pup brains of the SHD group, although the pups of the SHD group on day 21 after birth showed 25.3% EA in SHD and the erythrocyte membrane was 8.6% EA. A myelination of the rat brain began at about 14 days after birth. It was supposed that very small amounts of EA in the brains of pups of the SHD group were detected because the blood brain barrier is not yet formed by day 8 after birth. Lipids in the maternal diet were reported to affect the myelination of the rat brain [29]. Moreover, DHA was essential for myelination of the rat brain, and DHA deficiency during infancy delays brain development, while in aging a DHA deficiency accelerates deterioration of brain functions [30, 31]. While the ALA in SHD was only 0.1%, the synthesized n-3 PUFA from ALA moved from the dams into the pups via breast milk, and the n-3 PUFA was synthesized in body of pups. On day 82 after birth, no significant difference in the n-3 fatty acid levels in the brains of pups was observed between the groups (Table 8). Therefore, it was considered that DHA was synthesized in the bodies of the pups.

In one study, pregnant mice were fed a diet containing 21.5% TFA and 0.1% ALA, and the relationship between the fatty acid composition of the brain and behavioral development in 7-week-old mice was investigated [19]. The control diet contained 1.1% ALA and no detectable amount of TFA. The brain DHA level of the TFA group was lower than that of the control group. Reversal learning in the T-water maze was significantly slower in the TFA group compared with the control group. The authors of that study referred that future studies of the long-term effects of dietary TFA during the pre- and postnatal periods should measure behavioral development and neural function of brain. Souza et al. fed pregnant and lactating rats a diet containing 14.1% TFA and investigated the relationship between relative fatty acid composition in total lipids of the hippocampi of offspring and the spatial memories of young rats [32]. Although TFA was detected in the brains of pups immediately after birth, the effects of dietary TFA on brain development and function remain unclear. “The FAO/WHO joint meeting of fat and fatty acid in human nutrition,” held in Japan in 2008, set out the target to intake of TFA less than 1% of total energy intake [33]. Kawabata et al. pointed out that daily TFA intake could become higher than the predicted value if processed foods containing high levels of TFA were consumed, while the average intake of TFA in young women was lower than the WHO-recommended energy ratio (<1%) [34]. It is advisable to avoid especially high levels of TFA-containing foods during pregnancy and lactation.

In summary, we have shown that EA intake during pregnancy and lactation supplies EA to plasma, remains in the erythrocyte membrane of pups, and moves into the brain in early infancy. We have also shown that the plasma T-cho concentration in pups on day 82 after birth tended to be higher in the SHD group (*P* = 0.062). These data highlight the need for further studies which should examine in more detail the long-term effects of dietary TFA during the pre- and postnatal periods and measure behavioral development and neural function of brain.

## Figures and Tables

**Table 1:** Composition of the experimental diets^a^.

Ingredient	(g/100 g)
Casein	20.0
L-Cystine	0.3
Cornstarch	49.95
Sucrose	10.0
Oil^b^	10.0
Cellulose	5.0
Mineral mixture^c^	3.5
Vitamin mixture^c^	1.0
Choline bitartrate	0.25
*tert*-Butylhydroquinone	0.0014

Energy (kcal/g)	3.8
Protein energy ratio (%)	21.3
Fat energy ratio (%)	24.7
Carbohydrate energy ratio (%)	54.0

^a^Composition for all ingredients is given in grams per 100 g of diet.

^b^Oil: SOD (soybean oil is added in SOD), SHD (shortening is added in SHD).

^c^Mineral and vitamin mixtures were based on the AIN-93G formulation [8].

**Table 2:** Fatty acid composition of soybean-oil diet (SOD) and shortening diet (SHD).

Fatty acid	SOD	SHD
	%wt	%wt
Saturated		
12:0	n.d.	1.1
14:0	0.1	0.7
16:0	11.4	18.5
18:0	n.d.	6.1
20:0	0.4	0.3
Total	**11.9**	**26.7**

Monounsaturated		
18:1	28.9	40.6
Total	**28.9**	**40.6**

Polyunsaturated		
18:2 (n-6)	52.5	6.4
18:3 (n-3)	6.3	0.1
n-6/n-3	8.3	64
Trans fatty acid		
18:1 trans	n.d.	25.3
18:2 (n-6) trans	n.d.	0.8
Total	** n.d.**	**26.1**

Unknown	0.4	0.8

**Table 3:** Food intake, body weight, liver and perirenal fat tissue weight, and plasma lipid concentration in dams.

Group	SOD	SHD
Food intake		
Pregnant period (g/19 days)	436 ± 44.1	426 ± 23.7
Nursing period (g/21 days)	897 ± 12.6	911 ± 28.7
Final body weight (g) (on day 21 after delivery)	306 ± 20.7	302 ± 21.4
Liver weight (g/100 g BW)	3.89 ± 0.40	4.17 ± 0.26
Perirenal fat tissue weight (g/100 g BW)	0.76 ± 0.10	0.65 ± 0.12
Plasma lipid concentration		
Triacylglycerol (mg/mL)	83.0 ± 17.0	106 ± 54.8
T-Cholesterol (mg/mL)	61.6 ± 11.6	60.1 ± 6.8

Values are mean ± SD; *n* = 5. Student's *t-*test: significantly different at *P* < 0.05.

T-Cholesterol: total cholesterol.

Pregnancy period: from day 3 of pregnancy to delivery (on day 21 of pregnancy).

Nursing period: from delivery to weaning (pups were weaned at day 21 after birth).

**Table 4:** Fatty acid composition of stomach content in pups on day 8 after birth nursed by dams fed the soybean oil or shortening diet.

Fatty acid	SOD	SHD
Saturated	59.5 ± 4.12	53.7 ± 3.76
Monounsaturated	17.9 ± 1.08	29.8 ± 2.59*
18:1 trans	n.d.	11.6 ± 1.03**
Polyunsaturated		
n-6	20.8 ± 3.32	3.83 ± 0.57**
n-3	1.38 ± 0.52	0.07 ± 0.02**
n-6/n-3	15.1	54.7
Unknown	0.42	1.00

Values are means ± SD; *n* = 5. Student's *t*-test: *significantly different from the SOD group at *P* < 0.05. **Significantly different from the SOD group at *P* < 0.01.

**Table 5:** Body, liver, and perirenal fat tissue weights of pups nursed by dams fed the soybean oil or shortening diet.

Days after birth	*n*	Body weight (g)		Liver weight (g/100 g)		Perirenal fat tissue weight (g/100 g)	
		SOD	SHD	SOD	SHD	SOD	SHD
8♂	9	17.9 ± 0.90^e^	18.9 ± 1.42^e^	3.45 ± 0.15^b^	3.13 ± 0.26^b^	—	—
8♀	10	18.3 ± 1.15^e^	18.1 ± 1.24^e^	3.90 ± 0.50^ab^	4.00 ± 0.20^a^	—	—
21♂	5	58.6 ± 2.93^d^	64.1 ± 2.74^d^	4.10 ± 0.34^a^	4.36 ± 0.12^a^	0.37 ± 0.09^c^	0.41 ± 0.24^c^
21♀	5	58.8 ± 3.96^d^	59.9 ± 1.98^d^	4.33 ± 0.26^a^	4.43 ± 0.28^a^	0.32 ± 0.10^c^	0.30 ± 0.10^c^
47♂	5	253 ± 9.53^c^	244 ± 11.7^c^	3.43 ± 0.14^b^	3.46 ± 0.29^b^	1.04 ± 0.23^b^	1.14 ± 0.29^b^
47♀	5	206 ± 49.1^c^	213 ± 35.1^c^	3.41 ± 0.07^b^	3.35 ± 0.31^b^	0.93 ± 0.39^b^	0.95 ± 0.46^b^
68♂	5	380 ± 72.9^ab^	365 ± 80.0^ab^	2.62 ± 0.26^c^	2.68 ± 0.30^c^	1.87 ± 0.27^a^	1.80 ± 0.62^a^
68♀	5	242 ± 6.11^c^	253 ± 21.6^c^	2.86 ± 0.21^c^	2.83 ± 0.04^bc^	1.18 ± 0.28^b^	1.05 ± 0.21^b^
82♂	5	450 ± 44.7^a^	477 ± 28.2^a^	2.88 ± 0.54^bc^	3.02 ± 0.18^bc^	2.13 ± 0.93^a^	1.93 ± 0.63^a^
82♀	5	280 ± 28.4^bc^	291 ± 64.0^bc^	3.12 ± 0.64^bc^	2.72 ± 0.11^c^	2.14 ± 0.95^a^	1.84 ± 0.82^a^

Values are means ± SD. Values not sharing common superscript letters are significantly different at *P* < 0.05.

**Table 6:** Plasma lipid concentration of pups nursed by dams fed the soybean oil or shortening diet.

Days after birth	*n*	Triacylglycerol (mg/dL)		Total cholesterol (mg/dL)	
		SOD	SHD	SOD	SHD
8	19	116.3 ± 28.6^a^	131.3 ± 42.6^a^	91.4 ± 25.1^abc^	108.2 ± 15.2^ab^
21	10	122.3 ± 15.2^a^	95.2 ± 7.34^a^	121.7 ± 15.5^a^	95.9 ± 7.47^b^
47♂	5	89.8 ± 26.1^ab^	68.5 ± 7.09^b^	60.5 ± 8.55^bc^	46.4 ± 8.89^c^
47♀	5	82.2 ± 30.8^ab^	56.6 ± 14.3^b^	63.3 ± 11.4^c^	50.0 ± 15.9^c^
68♂	5	75.2 ± 13.8^b^	52.6 ± 11.7^b^	58.6 ± 11.1^c^	55.5 ± 9.18^c^
68♀	5	56.1 ± 12.8^b^	58.6 ± 10.1^b^	57.0 ± 15.3^c^	59.4 ± 15.7^c^
82♂	5	84.5 ± 12.2^b^	81.7 ± 26.4^ab^	67.7 ± 15.8^bc^	81.5 ± 12.3^bc^
82♀	5	65.9 ± 6.88^b^	94.2 ± 32.0^ab^	54.8 ± 13.2^c^	67.6 ± 13.2^bc^

Values are means ± SD. Values not sharing common superscript letters are significantly different at *P* < 0.05.

**Table:** (a)

Days after birth	8			
Group	Plasma		Blood cell membrane	
	SOD	SHD	SOD	SHD
*n*	9	10	9	10
Saturated	45.1 ± 3.46^a^	40.0 ± 2.12^b^	40.3 ± 1.90^b^	39.4 ± 1.74^b^
Monounsaturated	11.6 ± 1.98^c^	26.0 ± 2.54^a^	17.0 ± 2.93^b^	26.0 ± 2.72^a^
18:1 trans	n.d.^c^	7.18 ± 1.20^a^	n.d.^c^	5.82 ± 1.00^b^
Polyunsaturated				
n-6	39.8 ± 1.31^a^	24.8 ± 3.21^b^	36.1 ± 3.24^a^	24.7 ± 3.85^b^
n-3	3.33 ± 0.86^b^	1.85 ± 0.57^c^	5.15 ± 1.29^a^	2.72 ± 0.91^bc^
n-6/n-3	12.0	13.4	7.01	9.08
Unknown	0.17	0.17	1.45	2.36

**Table:** (b)

Days after birth	21			
Group	Plasma		Blood cell membrane	
	SOD	SHD	SOD	SHD
*n*	10	10	10	10
Saturated	31.9 ± 1.72^b^	30.5 ± 9.65^b^	39.1 ± 3.48^a^	36.9 ± 2.66^a^
Monounsaturated	13.1 ± 1.39^c^	29.1 ± 2.83^a^	17.5 ± 1.71^b^	28.1 ± 1.64^a^
18:1 trans	n.d.^c^	14.1 ± 1.80^a^	n.d.^c^	8.62 ± 1.46^b^
Polyunsaturated				
n-6	50.9 ± 1.12^a^	22.5 ± 2.66^c^	38.6 ± 3.62^b^	24.4 ± 3.43^c^
n-3	3.35 ± 0.83^b^	0.97 ± 0.50^c^	4.74 ± 0.96^a^	1.73 ± 0.31^c^
n-6/n-3	47.6	28.5	8.14	14.1
Unknown	2.56	2.83	0.06	0.25

Values are means ± SD. Values not sharing common superscript letters are significantly different at *P* < 0.05.

**Table 8:** Fatty acid composition of brains of pups, on days 8 and 21 after birth, nursed by dams fed the soybean oil or shortening diet.

Days after birth	8		21		82	
Group	SOD	SHD	SOD	SHD	SOD	SHD
*n*	9	10	10	10	10	10
Brain (g/100 g BW)	4.35 ± 0.40^a^	4.20 ± 0.28^a^	2.48 ± 0.29^b^	2.29 ± 0.27^b^	0.62 ± 0.16^c^	0.55 ± 0.14^c^
Total fat (mg/g brain)	12.9 ± 3.11^c^	15.1 ± 5.16^c^	34.0 ± 8.09^b^	34.3 ± 4.12^b^	65.3 ± 10.2^a^	65.2 ± 4.09^a^
Saturated	53.3 ± 3.06^a^	52.0 ± 1.23^ab^	49.1 ± 1.62^b^	54.1 ± 2.61^a^	45.3 ± 2.08^c^	45.5 ± 0.39^c^
Monounsaturated	17.5 ± 2.05^d^	19.7 ± 0.98^c^	20.3 ± 0.81^bc^	21.9 ± 1.24^b^	30.0 ± 0.72^a^	29.2 ± 0.95^a^
18:1 trans	n.d.^b^	0.56 ± 0.24^a^	n.d.^b^	n.d.^b^	n.d.^b^	n.d.^b^
Polyunsaturated						
n-6	16.9 ± 2.83^a^	18.6 ± 0.65^a^	17.4 ± 0.98^a^	15.7 ± 2.77^ab^	11.8 ± 1.06^c^	13.7 ± 1.16^b^
n-3	12.3 ± 0.38^a^	8.52 ± 0.47^b^	12.3 ± 0.38^a^	8.18 ± 1.15^b^	11.4 ± 0.76^a^	11.4 ± 0.62^a^
n-6/n-3	1.34	2.18	1.41	1.92	1.04	1.20
Unknown	0.00	0.62	0.90	0.12	1.50	0.20

Values are means ± SD. Values not sharing common superscript letters are significantly different at *P* < 0.05.
